# Epidermodysplasia verruciformis in a HIV patient – case report

**DOI:** 10.1186/1471-2334-14-S4-P16

**Published:** 2014-05-29

**Authors:** Anca Răducan, Adina Alexandru, Irina Magdalena Dumitru, Sorin Rugină

**Affiliations:** 1 Colentina Clinical Hospital, Bucharest, Romania; 2 Ovidius University, Constanța, Romania; 3 Clinical Hospital of Infectious Diseases, Constanța, Romania

## 

Epidermodysplasia verruciformis (EV) is a rare genodermatosis with an autosomal recessive pattern, characterized by an unique susceptibility to chronic cutaneous infections involving specific human papilloma virus (HPV) types in the genus β. Acquired EV, with clinical features similar to those in congenital EV, occurs only in immunocompromised patients, including those with HIV, lymphoma, transplant recipients and patients undergoing immunosuppressive therapy.

Patients frequently present with verruca plana-like lesions (discrete or confluent, often red-brown papules) distributed on the extremities, usually dorsal hands, face and neck. Other characteristic clinical findings include flat scaly pinkish, red-brown or hypopigmented guttate macules or thin plaques, which are similar to pityriasis versicolor, especially if they develop on the trunk. EV is considered a premalignant condition and almost half of the patients develop in the fourth and fifth decades squamous cell carcinoma, most commonly on sun-exposed area.

We report the case of a 26 years-old female patient with a history of HIV infection since the age of 6 years, receiving antiretroviral therapy, with a CD4 lymphocyte count of 545/μL and undetectable viral load, who presented for treatment of asymptomatic but cosmetically distressing skin lesions that had been present for almost 10 years.

Dermatological examination revealed isolated flat and some confluent, reddish-brown discrete papules and erythematous macules, 2-7 mm in diameter, distributed on the dorsal hands, forearms and knees.

Dermoscopy exhibited well circumscribed erythematous area with a whitish, scaly surface.

Histopathology showed hyperkeratosis, slightly thickened epidermis, enlarged keratinocytes, some with basophilic and others with eosinophilic cytoplasm, hypertrophic nuclei with perinuclear halos, and intracytoplasmic keratohyalin granules.

The diagnosis of EV verruca plana-like was confirmed. The patient was counseled about the disease and topical therapy with imiquimod cream was initiated, without improvement at one month follow-up. The patient is still on treatment and further therapeutic options are considered (cryotherapy, TCA peeling, electrotherapy and acitretin).

EV in HIV is a rare condition, with only 30 cases reported in medical literature, and this patient is the first case of EV-HIV coinfection in our HIV department.

